# Clergymen and Physicians

**Published:** 1856-05

**Authors:** 


The following article should be received with a reservation, that we should bestow no favors on that numerous class of the clergy who are the active supporters of quackery. Here, as elsewhere, discrimination is proper, and no general rule should be adopted.

*Clergymen and Physicians.* By THEOPHILUS PARVIN, M, D., of Indianapolis, Indiana.

The article with this title by an esteemed member of our profession, Dr. J. T. Plummer, of Richmond, in the last number of the *Observer*, by no means exhausts the question discussed, nor does it, in my mind at least, produce conviction of the justice of the side advocated by him.

Ought physicians as a rule, subject to exceptions of course, to render gratuitous service to clergymen?

Assuming the fact of a revelation by God to men in the Bible—vastly important to him here and hereafter, and the necessity of that revelation being expounded, its truths enforced and principles inculcated regularly by educated christian men, premises that we believe the great majority will concede—we unhesitatingly answer the question in the affirmative. Gratuitous medical services are, in some degree, a voluntary tribute to the worth and importance of the mission of ministers. But we do not rest our decision on this ground mainly.

In opposition to Dr. Plummer, the writer most certainly believes that there are "closer relationship, more natural ties, more proper civil attraction or attachment" between physicians and clergymen, than between the physician and any other class of men. Both are, or ought to be, completely educated men alike in literature and science; men of warm sympathies and of noble humanity; they meet at the bed of the sick and dying; each knows the frailty of human life and the uncertainty of human hopes, and none are so associated together in the minds of the family, none so precious to their hearts as the spiritual and the medical advisers are; where fitted in head and heart for their respective callings, they often can and do assist each other, and there will be both intellectual and social communion between them such as does not exist on the part of either, with any other outside of their respective professions.

"Are clergymen a privileged class among us, that they should exact gratuities from any? Are they themselves willing to labor in their vocation without the hope of reward?"

In reply, let me say that clergymen do not "*exact* gratuities" from us; we give them our advice and services voluntarily—they are not claimed by them so much as a *right*, as bestowed on our part from a generosity and nobleness which I trust will ever live among us. As to clergymen being unwilling to labor without a reward, I would say that a worse remunerated class of men, so far as dollars and cents are concerned, does not exist in our land, when we consider the amount of time and means invested in their preparation for their calling. For one who may be in the reception of "his thousands for his labor and faring sumptuously every day," there are twenty who do not receive five hundred; and surely, my respected friend has not seen the interior of many ministers' houses when he writes of "*faring sumptuously* every day."

Let me give a fact or two in reference to clergymen's salaries: The largest salary, probably, paid in this State to any minister, certainly the largest paid to any Presbyterian minister, is fifteen hundred dollars, and this sum was given to the Rev. John A. McClung, of this city; at the bar Mr. McClung's income would have been five times that amount. The average of the salaries of Presbyterian clergymen, the State over, is scarcely four hundred dollars, and this is a very liberal estimate. The same amount of education, energy and industry, invested in any other calling, would produce an average income at least triple in amount. Facts derived from other religious denominations would lead us to similar conclusions.

A clergyman, even with such a meagre pittance, has claims upon his hospitality and benevolence such as other men have not; his house is headquarters for traveling ministers, and agents of various descriptions, and his name must head all subscription papers for the poor and every other charity.

Moreover, he must expend a considerable portion of his income in replenishing his library, and in procuring scientific, literary, and theological journals, if he would keep abreast of the popular knowledge of the day and know what phases public opinion is taking, and how best, in the pulpit and in social intercourse, to combat the errors of the times, and most effectually to influence the minds and hearts of men. How all these demands are to be met, and a wife, with progeny sometimes as numerous as poor Mrs. John Rogers', to be supported, children clothed and educated, and doctor's bills paid, with four hundred dollars a year, especially when from the nature of the minister's avocation, if faithful in the discharge of his duties, he is excluded from common sources of emolument, is a problem I cannot solve. I know full well that there are hundreds of ministers who, from the day of their first settlement until the day of their death, are engaged in a ceaseless struggle to "keep the wolf from the door."

For one, then, I protest against a disturbance of the custom which has existed in this matter from time immemorial. Let us not compel clergy who may have our advice and attentions, to "confess" poverty, or else cause them to hamper their usefulness and make their families suffer, to settle our bills. The presumption ought always to be, unless facts of the opposite are decidedly apparent, a preacher cannot well pay a bill for medical services; no bill should be presented, no fee should be accepted from him unless we *know* that he is quite able to give that fee.

Every clergyman whom the writer knows, who receives a liberal income, pays his physician. This, I believe, will be found to be the case in nine cases out of ten. And even if we render hundreds of dollars in gratuitous professional services to ministers, I must believe that under the Divine economy, we will lose nothing ultimately.—*Cincinnatti Med. Observer.*

